# *Collyricloides massanae* (Digenea, Collyriclidae): spermatozoon ultrastructure and phylogenetic importance

**DOI:** 10.1051/parasite/2014061

**Published:** 2014-11-14

**Authors:** Abdoulaye Jacque Bakhoum, Yann Quilichini, Jordi Miquel, Carlos Feliu, Cheikh Tidiane Bâ, Bernard Marchand

**Affiliations:** 1 CNRS – University of Corsica, UMR SPE 6134, SERME “Service d’Étude et de Recherche en Microscopie Électronique” 20250 Corte France; 2 Laboratory of Evolutionary Biology, Ecology and Management of Ecosystems, Faculty of Sciences and Techniques, Cheikh Anta Diop University of Dakar BP 5055 Dakar Senegal; 3 Laboratori de Parasitologia, Departament de Microbiologia i Parasitologia Sanitàries, Facultat de Farmàcia, Universitat de Barcelona Av. Joan XXIII, sn 08028 Barcelona Spain; 4 Institut de Recerca de la Biodiversitat, Facultat de Biologia, Universitat de Barcelona Av. Diagonal 645 08028 Barcelona Spain

**Keywords:** *Collyricloides massanae*, Collyriclidae, Gorgoderoidea, Microphalloidea, Spermatozoon, Phylogeny

## Abstract

The spermatological characteristics of *Collyricloides massanae* (Digenea: Collyriclidae), a parasite of *Apodemus sylvaticus* caught in France, were studied by means of transmission electron microscopy. The mature sperm of *C. massanae* presents two axonemes of different lengths with the 9 + “1” pattern of the Trepaxonemata, two bundles of parallel cortical microtubules, external ornamentation of the plasma membrane, spine-like bodies, one mitochondrion, a nucleus and granules of glycogen. An analysis of spermatological organisation emphasised some differences between the mature spermatozoon of *C. massanae* and those reported in the Gorgoderoidea species studied to date, specially belonging to the families Dicrocoeliidae, Paragonimidae and Troglotrematidae. The ultrastructural criteria described in *C. massanae* such as the morphology of both anterior and posterior spermatozoon extremities, the association “external ornamentation + cortical microtubules”, the type 2 of external ornamentation and the spine-like bodies would allow us to bring closer the Collyriclidae to Microphalloidea. However, further ultrastructural and molecular studies are needed particularly in the unexplored taxa in order to fully resolve the phylogenetic position of the Collyriclidae.

## Abstract (fr)

Les caractères spermatologiques de *Collyricloides massanae* (Digenea : Collyriclidae) parasite d’*Apodemus sylvaticus* capturé en France, sont étudiés au microscope électronique à transmission. Le spermatozoïde mûr de *C. massanae* présente deux axonèmes de longueurs distinctes et de type 9 + « 1 » des Trepaxonemata, deux champs de microtubules corticaux parallèles, des ornementations externes, des corps épineux, une mitochondrie, un noyau et des granules de glycogène. Une analyse de l’organisation du spermatozoïde montre certaines différences entre le spermatozoïde mûr de *C. massanae* et ceux des espèces de Gorgoderoidea étudiées jusqu’ici, particulièrement celles appartenant aux familles Dicrocoeliidae, Paragonimidae et Troglotrematidae. Les critères ultrastructuraux décrits chez *C. massanae* tels que la morphologie des extrémités antérieure et postérieure, l’association « ornementation externe + microtubules corticaux », l’ornementation externe de type 2 et les corps épineux pourraient nous permettre de rapprocher les Collyriclidae des Microphalloidea. Cependant, des études ultrastructurales et moléculaires seront nécessaires, en particulier chez les taxa inexplorés, pour la résolution complète de la position systématique des Collyriclidae.

## Introduction

The Collyriclidae Ward, 1917 is a small family with two genera and two species namely *Collyriclum faba* (Bremser in Schmalz, 1831) and *Collyricloides massanae* (Vaucher, 1969) [56]. This family is distinguished from other members of the Gorgoderoidea by the location of adults in the host, i.e. cysts in the skin or intestinal wall of birds and mammals [8]. The monotypic genus *Collyricloides* described by Vaucher [56] was established for a species from cysts in the intestinal wall of rodents in France. The genus *Collyricloides* is mainly distinguished from *Collyriclum* by a well-developed cirrus-sac and the presence of a ventral sucker which is absent in *Collyriclum*.

The classification of Collyriclidae within the Gorgoderoidea is questionable. In fact, this family was included in Gorgoderoidea for convenience of identification, based purely on its morphological similarities to the families recognised by molecular studies such as Dicrocoeliidae, Paragonimidae, Troglotrematidae, etc. [9, 39]. In Heneberg and Literák [15] phylogenetic analysis based on an 18S rDNA sequence, the collyriclid *Collyriclum faba* was seen to be closely related to some species from the families Prosthogonimidae, Pleurogenidae and Microphallidae included in their analysis. Thus, according to these findings, the family Collyriclidae would be classed in the superfamily Microphalloidea (instead of Gorgoderoidea). Following the opinion of Heneberg and Literák [15], the Collyriclidae is placed in the Microphalloidea in this study.

In order to understand phylogenetic classification, the present contribution follows those produced in recent years in digenean spermatological studies [4–6, 20, 21, 26–32, 36–38, 41, 46–50]. This study also provides new approaches on digenean phylogenetic relationships by means of ultrastructural data as reported so far in Cestoda and Monogenea belonging to the Neodermata [16–18, 25]. We describe for the first time the spermatological characteristics of *Collyricloides massanae*. In addition, a comparative spermatological study is carried out in order to understand relationships within the Digenea in general, Gorgoderoidea and Microphalloidea in particular. Moreover, our results are compared with those from molecular studies.

## Materials and methods

Specimens of *Collyricloides massanae* were collected live from a naturally infected *Apodemus sylvaticus* (Linnaeus, 1758) caught in the Natural Reserve of Py (France). The worms were isolated from their hosts, fixed in cold (4 °C) 2.5% glutaraldehyde in 0.1 M sodium cacodylate buffer at pH 7.4, rinsed in 0.1 M sodium cacodylate buffer at pH 7.4, post-fixed in cold (4 °C) 1% osmium tetroxide in the same buffer for 1 h, rinsed in 0.1 M sodium cacodylate buffer at pH 7.4, dehydrated in an ethanol series and propylene oxide, embedded in Spurr resin and polymerised at 60 °C for 72 h. Ultrathin sections (60–90 nm) in the seminal vesicle were cut on an ultramicrotome (Power tome PC, RMC Boeckeler^®^). The sections were placed on 300 and 200 mesh copper grids and double-stained with uranyl acetate and lead citrate according to Reynolds [52]. The cytochemical test of Thiéry [55] was used to locate glycogen on gold grids. Finally, all sections were examined on a Hitachi H-7650 transmission electron microscope, operating at an accelerating voltage of 80 kV, in the “Service d’Étude et de Recherche en Microscopie Électronique de l’Université de Corse” (Corte, France).

## Results

From examination of cross- and longitudinal sections in the seminal vesicle of *Collyricloides massanae*, four distinctive regions are evidenced in the mature spermatozoon.

### Region I (Figs. 1 and 4I)

**Figure 1.**
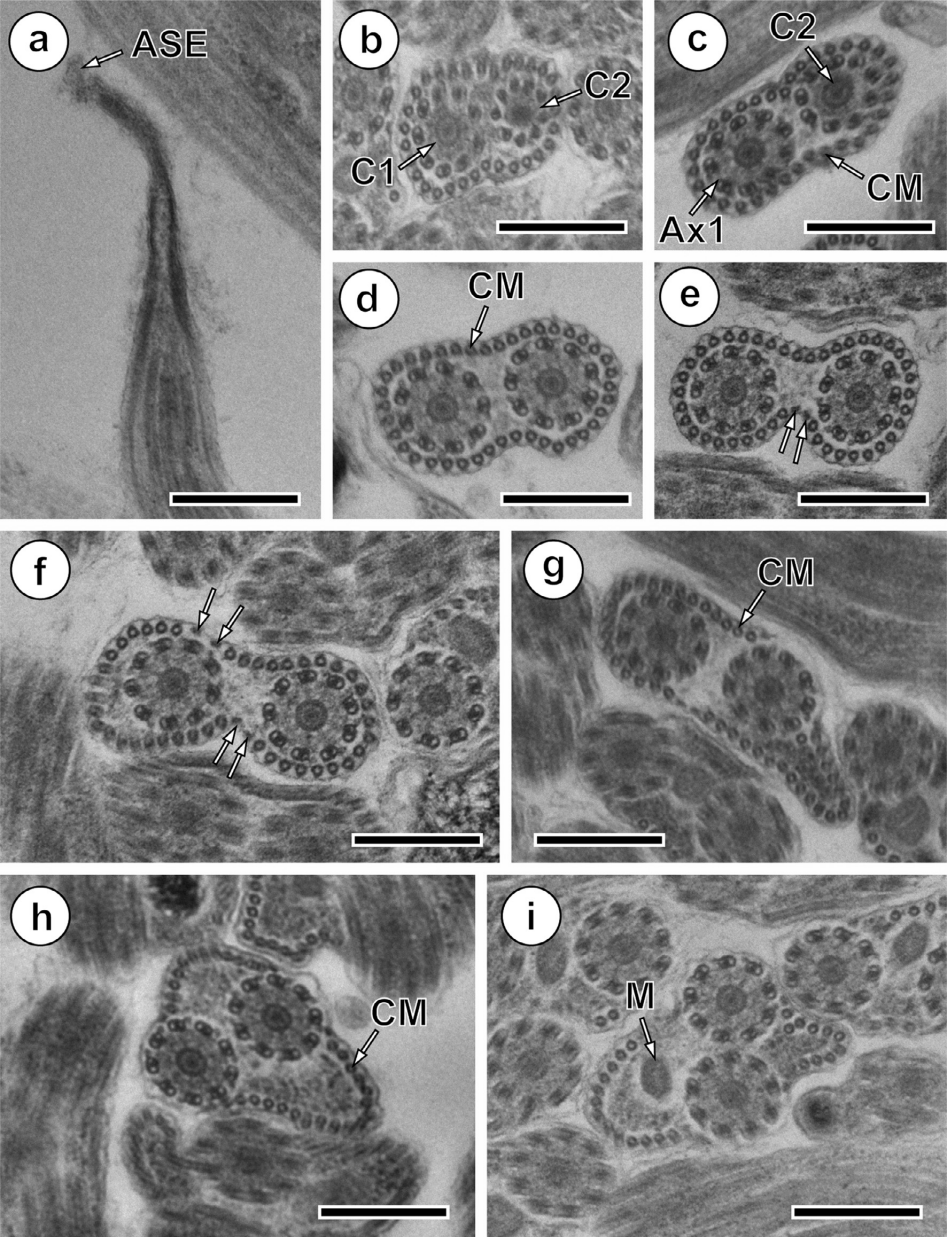
Mature spermatozoon of *Collyricloides massanae*. (a) Sharp morphology of the anterior spermatozoon extremity (ASE). (b, c) Cross-sections in Region I showing two centrioles (C1 and C2) corresponding to both axonemes and continuous layer of submembranous cortical microtubules (CM). Ax1, first axoneme. (d) Cross-section in which both axonemes are already formed and surrounded by a continuous layer of parallel cortical microtubules. (e, f) Consecutive cross-sections in the middle part of Region I exhibiting two and four attachment zones (arrows), interrupting the continuous layer of cortical microtubules. (g–i) Posterior part of Region I showing in cross-sections both axonemes and cortical microtubules organised into two fields separated by the four attachment zones. The appearance of the mitochondrion is also noticeable (M). Scale in μm: (a), 0.5; (b–i), 0.3.

Region I represents the anterior spermatozoon extremity showing in longitudinal section a sharp morphology (Figs. 1a and 4I). Cross-sections in the anterior tip exhibit centrioles of both axonemes surrounded by a continuous layer of parallel cortical microtubules of which the number varies from 31 (Fig. 1b) to about 33 when the first axoneme is formed (Figs. 1c and 4I). In addition, when both axonemes are completely formed the number of cortical microtubules is about 40 (Figs. 1d and 4I). Consecutive cross-sections in more posterior areas of Region I exhibit two axonemes also surrounded by a layer of cortical microtubules interrupted firstly by two attachment zones (Fig. 1e) and later by four attachment zones (Fig. 1f). Attachment zones delimit two fields of cortical microtubules and the maximum number of these begins to decrease from 42 to 41 (Figs. 1e−g and 4I). In the distal part of Region I, the cortical microtubules appear on either side of the axis formed by the two axonemes and their maximum number is about 29 in Fig. 1h and 23 in Fig. 1i. It is interesting to remark the appearance of the mitochondrion in the side containing the great number of cortical microtubules (Figs. 1i and 4I).

### Region II (Figs. 2a, b and 4II)

**Figure 2.**
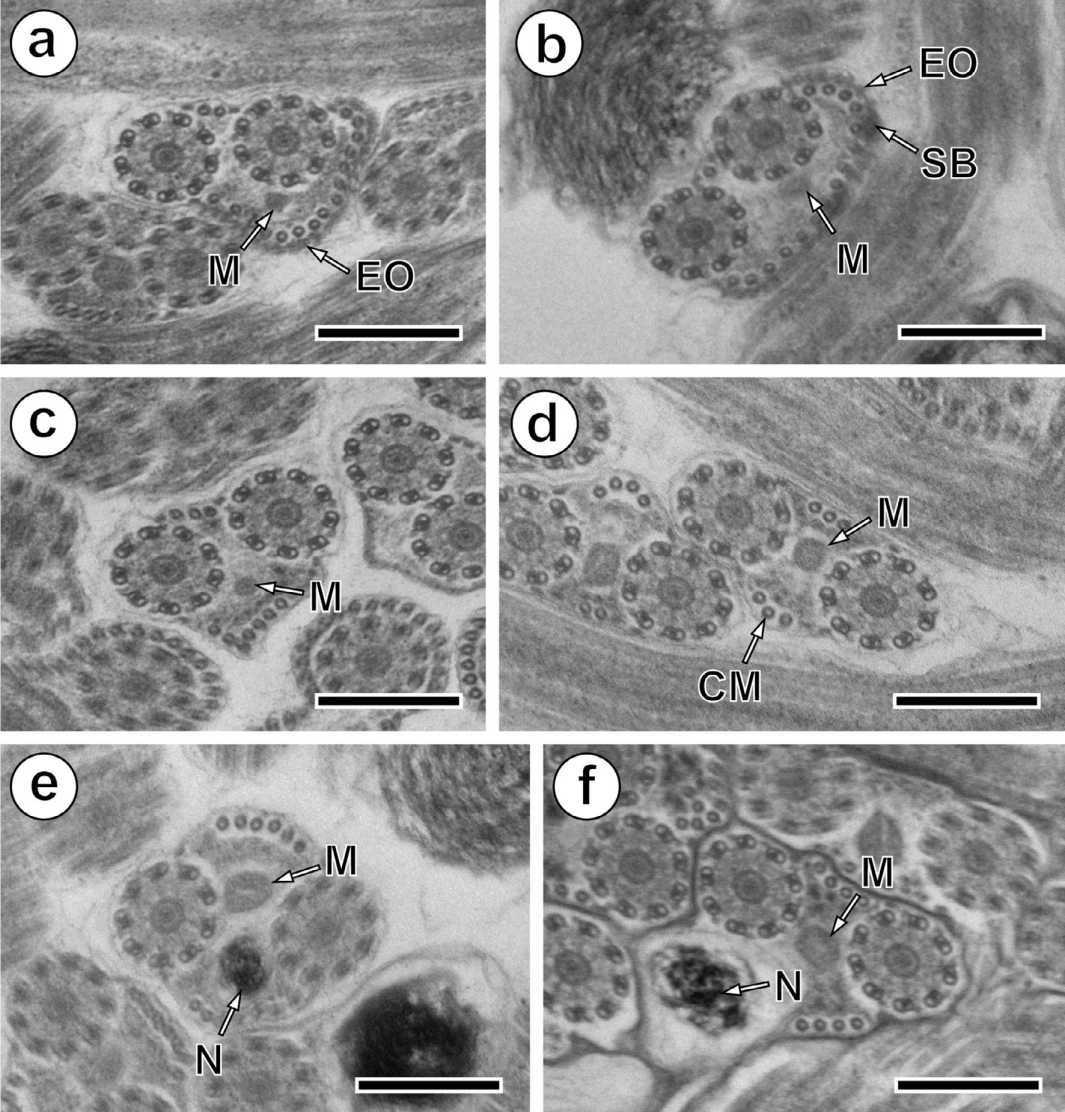
Mature spermatozoon of *Collyricloides massanae*. (a, b) Cross-sections in Region II or the ornamented zone of the mature spermatozoon exhibiting the mitochondrion (M), external ornamentation of the plasma membrane (EO) and spine-like body (SB). Note the association “external ornamentation + cortical microtubules”. (c, d) Region III or transitional area before nuclear appearance, exhibiting in cross-sections, only both axonemes, mitochondrion (M) and few cortical microtubules (CM). (e, f) Cross-sections in the proximal part of Region IV, showing the nucleus (N) accompanied by mitochondrion and cortical microtubules. Note the increase in size of the nucleus and its eccentric position. Scale in μm: 0.3.

Region II is an ornamented zone, characterised by the presence of external ornamentation of the plasma membrane associated with cortical microtubules and spine-like bodies. Both axonemes and mitochondrion are still present and the number of cortical microtubules is about 17–16 (Figs. 2a, b and 4II).

### Region III (Figs. 2c, d and 4III)

Region III corresponds to the transitional area before the nuclear region. It shows only two axonemes, mitochondrion, cortical microtubules and granules of glycogen. Moreover, a decrease of maximum number of cortical microtubules from 10 (Figs. 2c and 4III) to 6 (Figs. 2d and 4III) can be seen.

### Region IV (Figs. 2e, f, 3a−f, 4IV)

**Figure 3.**
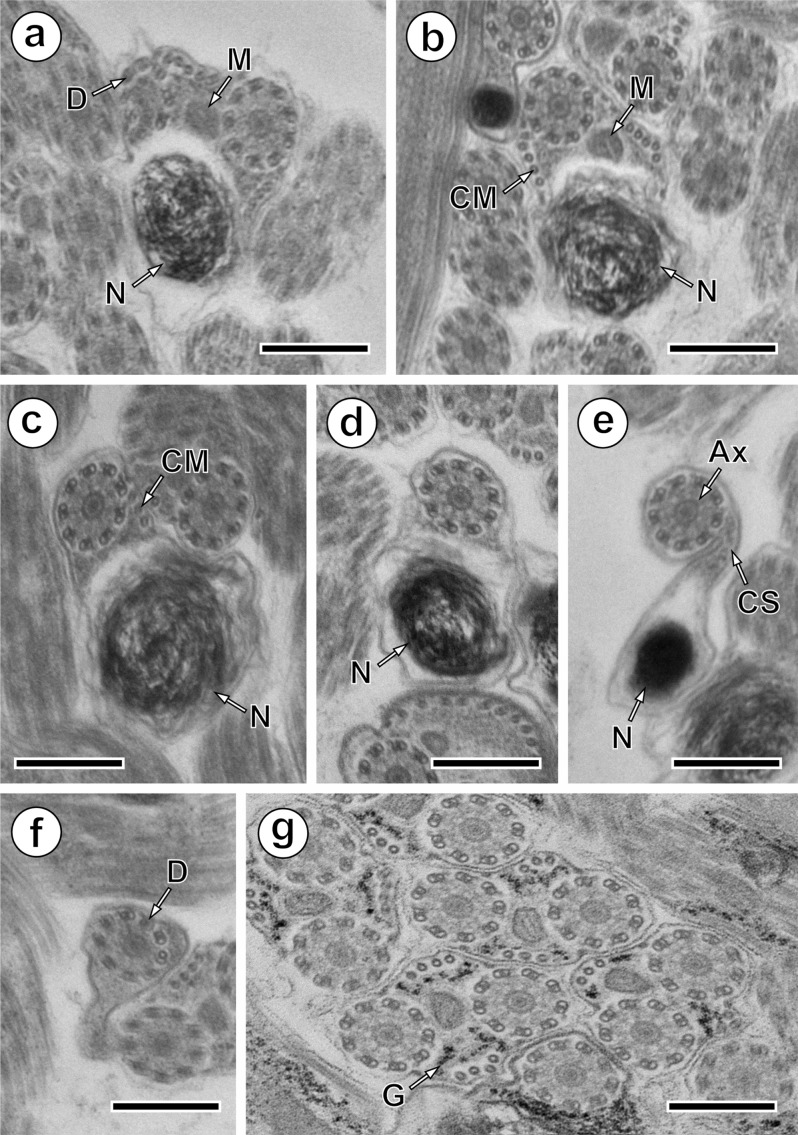
Mature spermatozoon of *Collyricloides massanae*. (a) Middle part of Region IV characterised by the disorganisation of the first axoneme into doublets (D). M, mitochondrion; N, nucleus. (b) Cross-section showing one axoneme, mitochondrion (M), nucleus (N) and cortical microtubules (CM). (c) Cross-section after mitochondrion disappearance exhibiting only nucleus (N), axoneme and few cortical microtubules (CM). (d, e) Consecutive cross-sections in the distal part of Region IV showing progressive decrease of nucleus diameter and appearance of “cytoplasmic stalk” (CS) between the nucleus (N) and the axoneme (Ax). Note also the disappearance of cortical microtubules. (f) Posterior spermatozoon tip with doublet (D) of microtubules. (g) Cross-sections showing the granules of glycogen (G) evidenced by Thiéry’s cytochemical test [51]. Scale in μm: 0.3.

**Figure 4.**
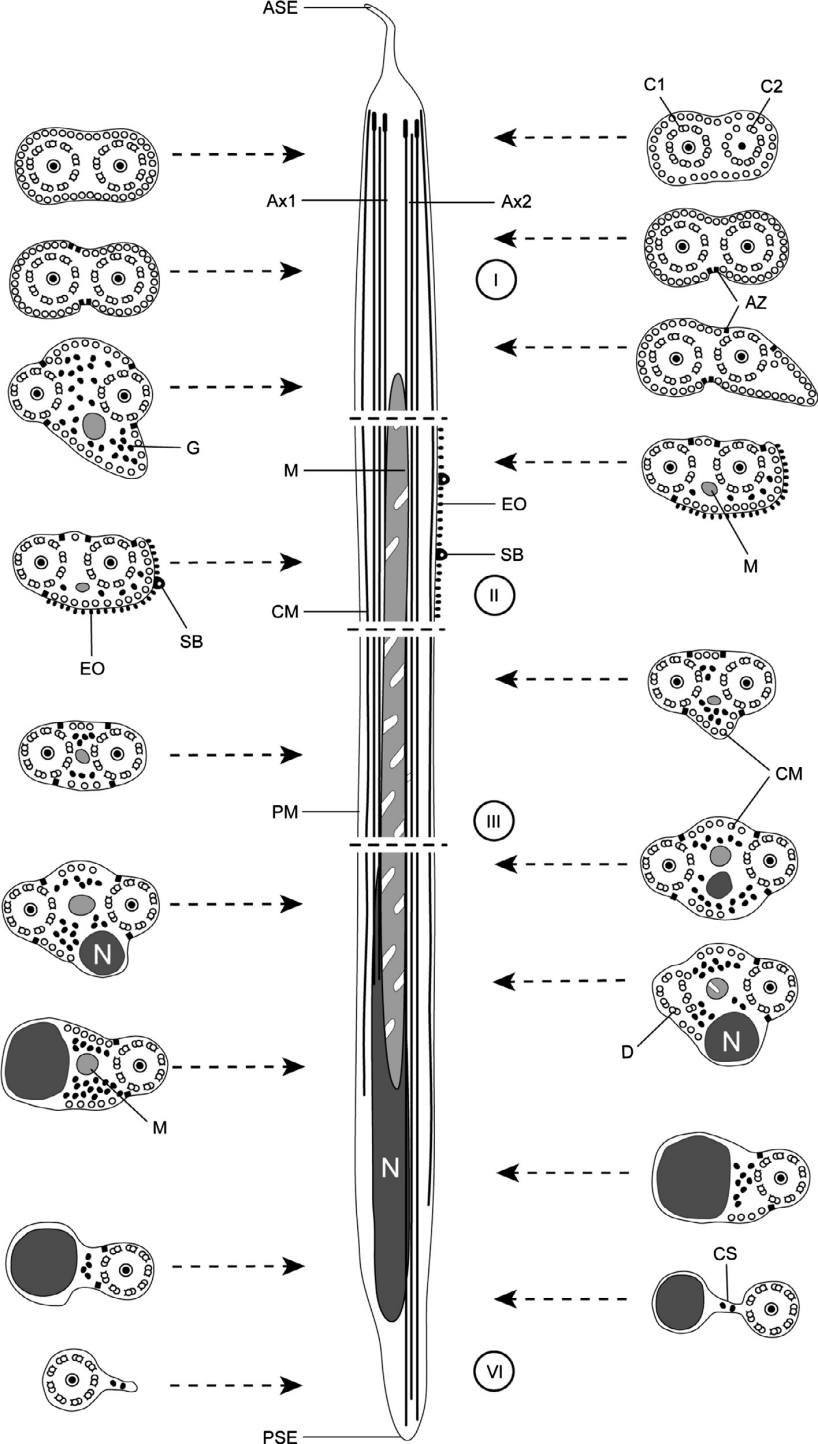
Schematic reconstruction of ultrastructural organisation of the mature spermatozoon of *Collyricloides massanae*. ASE, anterior spermatozoon extremity; Ax1, first axoneme; Ax2, second axoneme; AZ, attachment zones; C1, centriole of the first axoneme; C2, centriole of the second axoneme; CM, cortical microtubules; CS, cytoplasmic stalk; D, doublets; EO, external ornamentation of the plasma membrane; G, granules of glycogen; M, mitochondrion; N, nucleus; PM, plasma membrane; PSE, posterior spermatozoon extremity; SB, spine-like bodies.

Region IV is the nuclear region or the posterior spermatozoon extremity. In its proximal part, the nucleus is accompanied by both axonemes and a reduced number of cortical microtubules disposed in a nuclear (or dorsal) side with about 3–4, and in mitochondrial (or ventral) side with about 6 cortical microtubules (Figs. 2e and 4IV). When the diameter of the nucleus increases, cross-sections show four cortical microtubules in both dorsal and ventral sides (Figs. 2f and 4IV). The middle part of Region IV is characterised by the disappearance of the first axoneme. At this level, cross-sections exhibit the second axoneme, doublets of the first disorganised axoneme, the nucleus, the mitochondrion, granules of glycogen and a reduced number of cortical microtubules (about 8) (Figs. 3a and 4IV). In more posterior areas only the nucleus, mitochondrion and microtubules are observed (Figs. 3b and 4IV). Cross-sections after the mitochondrion disappearance show the nucleus, axoneme and a few microtubules (about 3) (Figs. 3c and 4IV). When microtubules disappear completely, cross-sections exhibit only the second axoneme and the nucleus with a diameter that reduces progressively (Figs. 3d, e and 4IV). Moreover, a cytoplasmic stalk appears between the nucleus and axoneme (Figs. 3e and 4IV). The posterior tip of the sperm cell is characterised by the disappearance of the nucleus and the presence of only the second axoneme (Figs. 3f and 4IV). Granules of glycogen have been evidenced by the cytochemical test of Thiéry (Fig. 3g).

## Discussion

### Spermatozoon general morphology

Ultrastructural characteristics described in *Collyricloides massanae* and most digenean spermatozoa could be classed into two types: (i) homogeneous or classical characters such as two axonemes of the 9 + “1” pattern of the Trepaxonemata, a nucleus, one or more mitochondria, cortical microtubules and granules of glycogen. These characters have been observed in all digenean species studied to date except those belonging to the Schistosomatidae and some Didymozoidae [19, 22, 23, 42]. On the other hand, (ii) several variable characters are present or absent according to digenean taxonomic levels (orders, superfamilies or families). Those are the external ornamentation of the plasma membrane, spine-like bodies, distribution of cortical microtubules into one or two parallel bundles, lateral expansion and morphology of both spermatozoon extremities [see 2]. In the present study, six ultrastructural characteristics are discussed below.

### Anterior spermatozoon extremity

The anterior spermatozoon extremity of *C. massanae* is formed by two slightly longitudinally displaced centrioles corresponding to both axonemes of the 9 + “1” trepaxonematan pattern. These two axonemes are surrounded by a continuous layer of cortical microtubules, observed in cross-section as a “corona”, the number of which is about 31 at the level of centrioles. This morphology differentiates mature spermatozoon of *C. massanae* from the other gorgoderoidean species studied until now (see Table 1). However, anterior spermatozoon morphology with two axonemes has been reported in other Digenea such as the Haematoloechidae *Haematoloechus* sp. [21], Microphallidae *Microphallus primas* [11], Monorchiidae *Monorchis parvus* [28], Omphalometridae *Rubenstrema exasperatum* [4], Pleurogenidae *Pleurogenes claviger*, *Pleurogenoides medians*, *Prosotocus confusus* [30] and *Brandesia turgida* [10] or the Prosthogonimidae *Mediogonimus jourdanei* [5].

**Table 1.** Ultrastructural characteristics of the mature spermatozoon in the Gorgoderoidea and Microphalloidea.

Taxa	MaxCM	ASE	LE	SB	CM + EO	TEO	M	TPSE	PSC
Gorgoderoidea									
Dicrocoeliidae									
*Dicrocoelium dendriticum* [12]	44	1Ax	−	−	−	?	1	3	1Ax
*Dicrocoelium hospes* [1]	36	1Ax	+	+	+	2	2*	2	N
*Corrigia vitta* [53]	33	1Ax	−	−	−	?	1	3	1Ax
Paragonimidae									
*Paragonimus ohirai* [41]	33	?	−	+	+	2	2	?	1Ax
Troglotrematidae									
*Troglotrema acutum* [30]	34	1Ax	+	+	+	2	2	3	1Ax
Microphalloidea									
Collyriclidae									
*Collyricloides massanae* (Present study)	42	2Ax	−	+	+	2	1	3	1Ax
Faustulidae									
*Pronoprymna ventricosa* [49]	8	1Ax	−	−	−	1	1	3	1Ax
Pleurogenidae									
*Brandesia turgida* [10]	41	2Ax	−	−	+	2	1	3	1Ax
*Pleurogenes claviger* [32]	43	2Ax	−	+	+	2	2*	3	1Ax
*Pleurogenoides medians* [32]	44	2Ax	−	+	+	2	2*	3	1Ax
*Prosotocus confusus* [32]	39	2Ax	−	+	+	2	2*	3	1Ax
Phaneropsolidae									
*Postorchigenes gymnesicus* [14]	36	?	−	−	+	2	2	3	1Ax
Prosthogonimidae									
*Mediogonimus jourdanei* [5]	40	2Ax	−	+	+	2	1	3	1Ax
Zoogonidae									
*Diphterostomum brusinae* [29]	39	1Ax	−	−	+	1	1	2	N

ASE, anterior spermatozoon extremity; Ax, axoneme; CM + EO, association “cortical microtubules + external ornamentation”; LE, lateral expansion; M, number of mitochondria; MaxCM, maximum number of cortical microtubules; N, nucleus; PSC, posterior spermatozoon character; TEO, type of external ornamentation location according to Quilichini et al. [48]; TPSE, type of posterior spermatozoon extremity according to Quilichini et al. [46]; SB, spine-like bodies; +/−, presence/absence of considered character; ?, unknown character.

* Presence of two mitochondria parallel in the mature spermatozoon.

Among the Gorgoderoidea studied until now, anterior spermatozoon extremities exhibiting one axoneme have been described in four species (see Table 1). With respect to the remaining species, lack of detail in their description does not allow us to give evidence of the morphology of their anterior spermatozoon extremities. It is interesting to remark that most digenean spermatozoa present only one axoneme in their anterior extremity. This is the case of species belonging to the families Brachylaimidae [35], Cryptogonimidae [13, 44, 54], Heterophyidae [3, 51], Opecoelidae [26, 31, 47] and Opistholebetidae [46].

Another type of anterior spermatozoon tip exhibiting only external ornamentation of the plasma membrane before appearance of centrioles is reported for example in the Apocreadiidae *Neoapocreadium chabaudi* [24]. Thus, variability concerning the morphology of the anterior spermatozoon extremity gives real evidence for the importance of this character in phylogenetic relationships in Digenea. It is interesting to note that determination of the morphology of the anterior extremity of the spermatozoon could be confusing in some species, in particular if both spermatozoon extremities contain one axoneme. In order to better describe the morphology of the anterior spermatozoon extremity, observation of sections showing centrioles is important, as is the case in *Collyricloides massanae*. Other spermatological characteristics such as the “presence/absence” of cortical microtubules and granules of glycogen would also be useful in the determination of the morphology of the anterior spermatozoon extremity.

### Presence, number and distribution of cortical microtubules

Cortical microtubules are present in the mature spermatozoon of all digenean species described to date, except those belonging to species of schistosomes and some didymozoids [19, 22, 23, 42]. In the mature spermatozoon of *Collyricloides massanae*, the maximum number of cortical microtubules is about 42 and located in the anterior spermatozoon extremity before appearance of mitochondrion and external ornamentation. Then, the number of cortical microtubules decreases from the anterior to the posterior spermatozoon extremity.

A similar arrangement of cortical microtubules has been reported in the Gorgoderoidea with a maximum number of about 37 in the Dicrocoeliidae [1, 12, 53], about 33 in the Paragonimidae [41] and about 34 in the Troglotrematidae [30]. Within the Microphalloidea, the maximum number of cortical microtubules varies within small margins (36–44), except in *Pronoprymna ventricosa* [49] in which 8 cortical microtubules were reported (see Table 1). The presence of two parallel bundles of cortical microtubules is observed in the mature spermatozoon of *C. massanae*. This characteristic has also been observed in all gorgoderoidean and most microphalloidean species studied until now [see 2]. In contrast, in members of certain digenean families only one field of cortical microtubules has been described. This is the case of the faustulid *Pronoprymna ventricosa* [49], the hemiurids *Lecithocladium excisum* and *Parahemiurus merus* [33, 34] and the lecithasterid *Aponurus laguncula* [45]. Therefore, the importance of “presence/absence” and number and arrangement of cortical microtubules would be interesting criteria in comparative analysis of Digenea and should be given more attention in future studies.

### Association “cortical microtubules + external ornamentation”

The association “cortical microtubules + external ornamentation” is observed in the anterior areas of the mature spermatozoon of *Collyricloides massanae* in which the external ornamentation is located on the mitochondrial side corresponding to the side with a great number of cortical microtubules (about 15). Among the Gorgoderoidea, the association “cortical microtubules + external ornamentation” is present in all species studied except two, namely *Corrigia vitta* and *Dicrocoelium dendriticum* [12, 53] (see Table 1). Nevertheless, in these studies, it is interesting to remark the absence of several micrographs concerning mature spermatozoon, especially those of the anterior spermatozoon area. In the microphalloidean species, the association “cortical microtubules + external ornamentation” is reported in all species except in *Pronoprymna ventricosa* [49] (see Table 1).

Numerous digenean species exhibit the association “cortical microtubules + external ornamentation” in their spermatozoa [2]. To emphasise the usefulness of external ornamentation of the plasma membrane for future application in phylogenetic analysis within the Digenea, Quilichini et al. [48] have established three morphological types according to external ornamentation location. The spermatozoon of *C. massanae* is of type 2, i.e. presence of external ornamentation at a distal area of the anterior spermatozoon extremity, usually in the mitochondrial region. This is also the case of the Gorgoderoidea such as *Dicrocoelium hospes*, *Paragonimus ohirai* and *Troglotrema acutum* [1, 30, 41], most microphalloidean species (see Table 1) and many other digenean spermatozoa [4, 5, 7, 24, 46].

The association “cortical microtubules + external ornamentation” and the variability in the location of external ornamentation of the plasma membrane could be very useful for the establishment of spermatozoon models and for phylogenetic purposes in Digenea.

### Spine-like bodies

Spine-like bodies have been described until now only in digenean species [2]. They are present in the anterior areas of the spermatozoon usually associated with external ornamentation of the plasma membrane.

In the mature spermatozoon of *C. massanae*, spine-like bodies are observed in the ornamented area associated with cortical microtubules and external ornamentation. Concerning the gorgoderoidean species studied until now, spine-like bodies have been described in three species (see Table 1). Their absence in some species such as *Corrigia vitta* or *Dicrocoelium dendriticum* would be interpreted as an omission. In fact, since the first description of spine-like bodies [31], these structures appear frequently in digenean spermatozoa, especially in the Gorgoderoidea in which all studies, after the year 2000, have mentioned the presence of spine-like bodies (see Table 1). In addition, spine-like bodies are clearly visible in micrographs (Figs. 6 and 7 in [41]) of the mature spermatozoon of the paragonimid *P. ohirai* [41]. However, in this study they are not mentioned, but were probably misinterpreted or considered as artefacts. The same is also likely in other digenean species, such as *Haematoloechus* sp. [21]. However, it is interesting to note the absence of spine-like bodies reported in some microphalloidean species (see Table 1) and species belonging to the families Brachylaimidae [35], Hemiuridae [33, 34], Lecithasteridae [45] and Sclerodistomidae [39]. Ndiaye et al. [39] interpreted the absence of spine-like bodies in the three latter families belonging to the Hemiuroideans as a plesiomorphy for this superfamily.

Although more ultrastructural studies are needed to confirm their real importance in digenean spermatozoa, spine-like bodies would be useful in comparative and phylogenetic studies at the family, superfamily or order levels.

### Variability in the number of mitochondria

The mature spermatozoon of *C. massanae* contains one mitochondrion. It appears before the ornamented area and reaches the nuclear region. Within the gorgoderoids and microphalloids, one mitochondrion is also described in the mature spermatozoon of *Corrigia vitta*, *Dicrocoelium dendriticum*, *Diphterostomum brusinae*, *Mediogonimus jourdanei* and *Pronoprymna ventricosa* (see Table 1), whereas in the remaining species, two mitochondria are evidenced in the mature spermatozoon (see Table 1). Based on logical interpretations of several cross-sections in the disposition of ultrastructural characteristics along the mature spermatozoon, the presence of more than one mitochondrion is now strongly evidenced in Digenea. Moreover, a parallel disposition of mitochondria is observed in some microphalloid species such as *D. hospes* [1], *Pleurogenes claviger*, *Pleurogenoides medians* and *Prosotocus confusus* [32].

As the absence of mitochondrion is considered a synapomorphy for the Eucestoda [16], we agree that the presence of at least one mitochondrion in digenean spermatozoa is a plesiomorphic character.

### Posterior spermatozoon extremity

Another interesting and variable criterion is the morphology of the posterior spermatozoon extremity. In *C. massanae*, the posterior part of the spermatozoon is ended by one axoneme. In fact, successions of ending characters towards the posterior tip allow us to establish this sequence: disappearance or absence of cortical microtubules, posterior extremity of the nucleus then posterior extremity of second axoneme. This latter sequence corresponds to type 3 or cryptogonimidean type according to Quilichini et al. [46]. Taking into account the postulated proximity of Collyriclidae to the Microphalloidea [15], it is important to remark that type 3 of the posterior spermatozoon extremity has also been reported in seven of the eight microphalloidean species studied until now (Table 1).

Among the gorgoderoids studied, posterior spermatozoon tips of type 3 were reported in the dicrocoeliids *C. vitta* and *D. dendriticum* [12, 53], the paragonimid *P. ohirai* [41] and the troglotrematid *T. acutum* [30]. With respect to the remaining species, posterior spermatozoon tips ended by the nucleus, corresponding to type 2 according Quilichini et al. [46], were observed in *D. hospes* [1].

A peculiar character observed in the posterior spermatozoon extremity of *C. massanae* is the presence of structure like a “cytoplasmic stalk”. A similar structure has also been described in the mature spermatozoon of the brachycoeliid *B. salamandrae* by Bakhoum et al. [6]. It is interesting to note that the Brachycoeliidae, previously included in Gorgoderoidea [9], have been moved to the Plagiorchioidea [see 40, 43]. Structures like a “cytoplasmic stalk” have also been reported in *N. chabaudi* [25] belonging to the Apocreadiidae. For this structure, no significant phylogenetic importance is evidenced.

As stated above for the anterior spermatozoon tip, the interest and variability of posterior spermatozoon morphology would be a valuable character when distinguishing digenean spermatozoa and would also be useful for establishing spermatozoon models.

### Phylogenetic approaches

Ultrastructural organisation of the mature spermatozoon of *Collyricloides massanae* emphasises some main characteristics useful for the analysis of phylogenetic affinities. Those are (i) the anterior spermatozoon extremity showing two axonemes, (ii) the presence of the association “cortical microtubules + external ornamentation”, (iii) type 2 external ornamentation, (iv) the presence of spine-like bodies and (v) the posterior spermatozoon extremity exhibiting one axoneme. These five ultrastructural characteristics observed in *C. massanae* are also reported in most microphalloidean species, especially those belonging to the families Pleurogenidae and Prosthogonimidae [5, 10, 32]. These similarities would allow us to support the close relationships between Collyriclidae and Pleurogenidae or Prosthogonimidae. Our ultrastructural findings therefore support inclusion of Collyriclidae in Microphalloidea, instead of Gorgoderoidea, as suggested by Heneberg and Literák [15]. However, further studies are needed considering that Gorgoderoidea were seen as a polyphyletic assemblage [15] and also considering the lack of studies in several of its families, particularly the type-family Gorgoderidae. Moreover, in several ultrastructural studies, a single genus has been explored for a family as in the present study.
